# Dopamine Precursor Depletion Influences Pain Affect Rather than Pain Sensation

**DOI:** 10.1371/journal.pone.0096167

**Published:** 2014-04-23

**Authors:** Laura Tiemann, Henrik Heitmann, Enrico Schulz, Jochen Baumkötter, Markus Ploner

**Affiliations:** 1 Department of Neurology, Technische Universität München, Munich, Germany; 2 TUM-Neuroimaging Center, Technische Universität München, Munich, Germany; 3 Department of Pediatrics, Technische Universität München, Munich, Germany; Federal University of Rio Grande do Norte, Brazil

## Abstract

Pain is a multidimensional experience, which includes sensory, cognitive, and affective aspects. Converging lines of evidence indicate that dopaminergic neurotransmission plays an important role in human pain perception. However, the precise effects of dopamine on different aspects of pain perception remain to be elucidated. To address this question, we experimentally decreased dopaminergic neurotransmission in 22 healthy human subjects using Acute Phenylalanine and Tyrosine Depletion (APTD). During APTD and a control condition we applied brief painful laser stimuli to the hand, assessed different aspects of pain perception, and recorded electroencephalographic responses. APTD-induced decreases of cerebral dopaminergic activity did not influence sensory aspects of pain perception. In contrast, APTD yielded increases of pain unpleasantness. The increases of unpleasantness ratings positively correlated with effectiveness of APTD. Our finding of an influence of dopaminergic neurotransmission on affective but not sensory aspects of phasic pain suggests that analgesic effects of dopamine might be mediated by indirect effects on pain affect rather than by direct effects on ascending nociceptive signals. These findings contribute to our understanding of the complex relationship between dopamine and pain perception, which may play a role in various clinical pain states.

## Introduction

Pain is a complex and highly subjective sensation involving sensory, cognitive, and affective aspects [1]. Converging lines of evidence suggest that dopaminergic neurotransmission plays an important role for the processing and perception of pain [2], [3]. Many parts of the central nervous system implicated in the processing of pain have a high density of dopamine receptors whose activation can yield analgesic effects in humans and experimental animals [4], [5]. Additionally, a dopaminergic influence on pain perception can be inferred from observations in dopamine-related neuropsychiatric disorders such as Parkinson’s disease and schizophrenia. In Parkinson’s disease, which is characterized by an abnormal decrease in dopaminergic transmission, pain is the most frequent non-motor symptom [6], [7]. Moreover, some studies showed that patients with Parkinson’s disease display a higher sensitivity to pain and greater brain responses to pain, which can be attenuated by an enhancement of dopaminergic neurotransmission [8], [9]. In contrast, in patients with schizophrenia, an insensitivity to pain has been documented early [10] and confirmed in more recent experimental studies [11], [12]. Further evidence for a dopaminergic influence on pain perception derives from observations of altered dopaminergic neurotransmission in various chronic pain conditions [13]–[16]. In addition, small clinical studies indicated that dopaminergic agents can relieve chronic pain [17]–[19]. Consequently, dopamine has been proposed to represent a potential therapeutic target in chronic pain syndromes [3], [20].

Taken together, experimental and clinical evidence suggests that dopamine can yield analgesic effects. It has been hypothesized that these pain modulatory effects of dopamine are mediated by direct effects on ascending nociceptive signals and/or by indirect effects on cognitive and affective aspects of pain [20]. However, no direct comparison of the effects of dopamine on sensory, cognitive and affective aspects of pain has been presented so far. Here, we therefore characterized the influence of dopaminergic neurotransmission on different aspects of pain perception in healthy human subjects. We applied Acute Phenylalanine and Tyrosine Depletion (APTD) as a non-invasive method to transiently reduce the cerebral dopamine level, and studied its effects on human pain perception and pain processing by means of electroencephalography (EEG).

## Methods

### Subjects

28 healthy male subjects participated in the study. Exclusion criteria included smoking, regular use of medication and a history of neurological or psychiatric disorders as assessed by an unstructured interview. Six subjects did not complete the study. Four subjects withdrew due to regurgitation of part of the amino acid mixture, one subject was excluded due to a vasovagal response following the blood draw, and one subject withdrew for personal reasons. Thus, analysis included data of 22 subjects with a mean age of 25 years (range 20–39 years). Procedures were approved by the local ethics committee (Ethikkommission der Technischen Universität München) and conducted in conformity with the declaration of Helsinki. Written informed consent was obtained from all subjects before participation.

### Procedure

**Figure 1:**
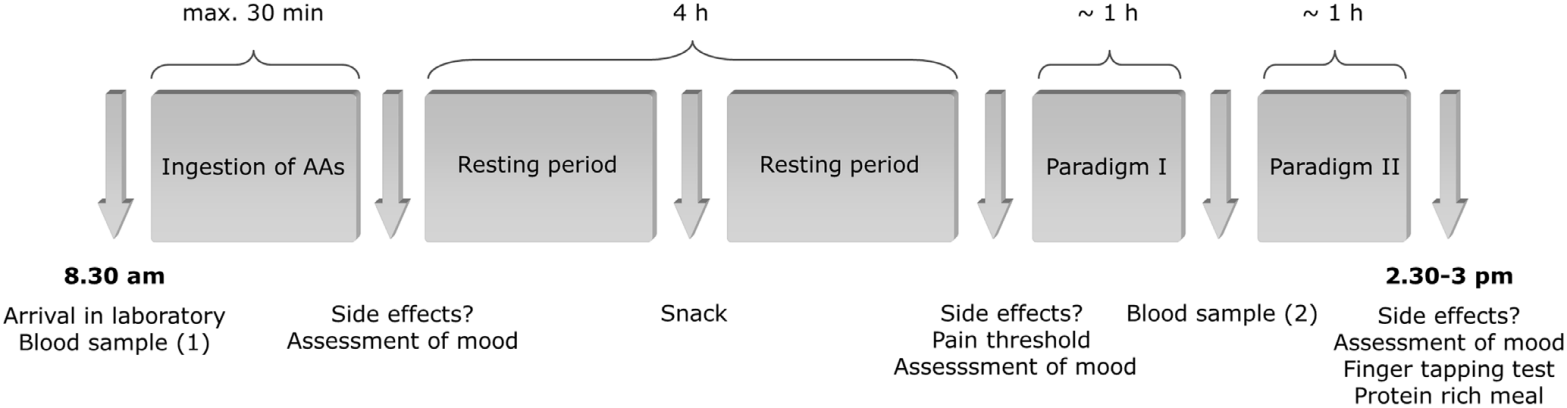
Timeline of events. Testing procedures were repeated on two days which were separated by one week. On one of these days, we applied Acute Phenylalanine and Tyrosine Depletion (APTD). The other day served as a control condition, on which the subjects ingested a balanced control mixture containing all essential amino acids.

Participants made two visits to the laboratory, on which they completed the exact same testing procedure (Fig. 1). On one of these days, we applied Acute Phenylalanine and Tyrosine Depletion (APTD), whereas the other day served as control condition. Testing days were separated by one week and order of conditions was balanced across subjects. Both investigators as well as subjects were blinded to the current experimental condition.

### Acute Phenylalanine and Tyrosine Depletion (APTD)

In APTD, selective dietary restriction of precursor amino acids (AA) needed for dopamine (DA) synthesis is used to transiently decrease DA neurotransmission in the human brain [21]. Participants ingest an AA mixture that either does or does not contain phenylalanine and tyrosine. The lack of phenylalanine and tyrosine does not yield any noticeable difference in taste, scent, or appearance of the AA mixture. Here, established amino acid compositions were used [22], with a depletion mixture (APTD) lacking the DA precursors tyrosine and phenylalanine, and a balanced control mixture (BAL) containing all essential amino acids. The procedure, thus, yields a relative deficit of dopaminergic neurotransmission in the APTD condition as compared to the BAL control condition [21].

The day prior to testing, subjects received a low protein diet (<10 g of protein; Loprofin Products, Heilbronn, Germany) and were not allowed to consume alcohol, caffeine and analgesics. Additionally, subjects had to fast from midnight with the exception of water consumption. On testing days, subjects arrived at the laboratory at 8∶30 am. A blood sample (10 ml) for evaluation of baseline AA levels was obtained. Mixtures were then prepared by dissolving amino acids in approximately 400 ml of water. In order to make the drink more palatable, black current or elder blossom syrup was added. Due to their unpleasant taste, the AAs Methionine, Cysteine, and Arginine were administered separately in capsules. Participants were given 30 minutes to ingest the amino acid mixture. This was followed by a resting period of 4 hours. Two hours post ingestion, subjects were offered a snack consisting of 2 slices of low-protein bread with honey. Testing was performed 4–6 hours post ingestion. The testing interval was chosen to coincide with maximum effects from APTD [21]. Five hours post ingestion, a second blood sample was drawn for evaluation of depletion effects on blood amino acid levels. On completion of the testing session, subjects performed a finger tapping test (INC Research, Raleigh, USA). Tapping rates were obtained for the forefinger of the dominant hand in three consecutive trials of 20 seconds each. Finally, subjects were offered a protein-rich meal. Side-effects were monitored using a 7-item questionnaire that included headache, dizziness, nausea, dry mouth, dry skin, blurred vision, and physical sluggishness at 30 minutes, 4 hours, and 6 hours post ingestion. Additionally, participants were instructed to report any side effects that were not listed. Subjects also rated their current mood on a 16-item questionnaire consisting of visual analogue scales with opposing verbal descriptors [23]. Mood was rated at 30 minutes, 4 hours and 6 hours post ingestion. Values were log-transformed and skewed scores were reversed. Next, scales were grouped into three categories: ‘alertness’ (ranging from ‘alert’ to ‘drowsy’), ‘calmness’ (ranging from ‘relaxed’ to ‘tensed’) and ‘contentment’ (ranking from ‘contented’ to ‘discontented’) [23].

Serum levels of AA were determined to assess the effectiveness of the depletion procedure [22]. Blood samples were centrifuged immediately and serum was fractioned-off and stored at −40°C until analysis. Serum levels of phenylalanine, tyrosine, and the other Long Neutral Amino Acids (LNAA) were measured using high-performance liquid chromatography (HPLC). Brain availability of tyrosine and phenylalanine was assessed by calculating ratios of tyrosine and phenylalanine serum levels to LNAA as previously described [22]. The tyrosine/LNAA ratio can be regarded as crucial marker for the efficiency of the depletion procedure, as the conversion of tyrosine to L-DOPA represents the rate-limiting step in DA synthesis [21].

### Paradigms

Pain thresholds to cutaneous laser stimulation were obtained using the method of limits at 4 hours post ingestion of AA mixtures on each of the testing days. Subsequently, we applied two paradigms to selectively characterize the influence of APTD on sensory, affective, and cognitive aspects of pain perception. Paradigm 1 assessed single trial pain intensity ratings as a sensitive measure of pain sensation [24]. Paradigm 2 evaluated cognitive and affective aspects of pain perception by means of a well-established visual-attention task [25], [26]. Order of paradigms was balanced across subjects.

#### Paradigm 1

75 brief painful laser stimuli were delivered to the dorsum of the right hand. Interstimulus intervals were randomly varied between 8 and 12 seconds. Three seconds after stimulus application, the subjects were prompted by an auditory cue to rate the pain intensity on a numerical rating scale between 0 (no pain) and 10 (maximum tolerable pain). Prior to the experiment, subjects were informed that pain intensity might vary during the experiment. However, all laser stimuli were of identical intensity (600 mJ). Subjects were exposed to white noise through headphones to cancel out noise of the laser device. The subjects perceived the stimuli with closed eyes.

#### Paradigm 2

To investigate the influence of APTD on a cognitive aspect of pain perception, participants completed an attention-demanding visual reaction time task with interfering painful laser stimulation. For further details of the paradigm please refer to [25]–[27].

Prior to the experiment, stimulus intensity was individually adjusted to match a rating of 5 on a numerical rating scale ranging from 0 (“no pain”) to 10 (“worst tolerable pain”). This resulted in statistically comparable objective stimulus intensity in the BAL (M ± SD; 440±87 mJ) and APTD (470±111 mJ) condition (t = 1.2, p = .2).

To investigate the influence of APTD on sensory and affective aspects of pain perception, subjects were asked to rate both the intensity and unpleasantness of the painful stimuli on completion of the task. To this end, a visual analogue scale ranging from 0 (“no pain” or “not unpleasant”, respectively) to 10 (“worst tolerable pain” or “highly unpleasant”, respectively) was used.

### Stimuli

Painful stimuli were applied to the dorsum of the hand using a Tm:YAG laser (Starmedtec GmbH, Starnberg, Germany) with a wavelength of 1960 nm, a pulse duration of 1 ms and a spot diameter of 5 mm. A distance pin mounted to the hand piece of the laser device ensured a constant distance between skin surface and laser device. Stimulation site was slightly varied after each stimulus to avoid tissue damage.

### EEG Recordings and Pre-processing

EEG data were recorded with an electrode cap (Easycap, Herrsching, Germany) and BrainAmp MR plus amplifiers (Brain Products, Munich, Germany) using the BrainVision Recorder software (Brain Products, Munich, Germany). Electrode montage included 64 scalp electrodes. Two more electrodes were fixed below the outer canthi of the eyes. The EEG was referenced to the FCz electrode, grounded at AFz, sampled at 1000 Hz and highpass-filtered at 0.1 Hz. The impedance was kept below 20 kΩ.

EEG data were preprocessed using the BrainVision Analyzer software (Brain Products, Munich, Germany). Offline analysis included downsampling to 512 Hz, digital highpass filtering at 0.5 Hz and recomputation to the average reference. Downsampling included automatic lowpass filtering at 230 Hz. Independent component analysis was used to correct for vertical and horizontal eye movements. Trials with artifacts exceeding ±100 µV in any channel were automatically rejected.

### Data Analysis

In paradigm 1 and 2, neurophysiological data of three subjects and one subject, respectively, had to be excluded from data analysis due to poor data quality. However, behavioral results remained unchanged after excluding these data sets from the behavioral analyses.

#### Paradigm 1

In paradigm 1, we assessed the effects of APTD on single trial intensity ratings of painful stimuli as a measure of sensory aspects of pain. Mean pain ratings and standard deviations were compared between APTD and BAL conditions. Habituation was evaluated by computing a regression line for the pain ratings in each condition and statistically comparing their slopes.

In order to assess the effects of APTD on laser-evoked potentials (LEP), data were segmented from −1000 to 2000 ms with respect to the painful laser stimulation and averaged using BESA 5.2. Amplitudes of LEP at all timepoints were compared between conditions (APTD and BAL). To control for type I error, false discovery rate (FDR) correction was performed across all electrodes and timepoints [28] using MATLAB (The Mathworks Inc., Natick, USA). Additionally, N2P2-peak-to-peak amplitudes were determined for every subject using the BrainVision Analyzer software (Brain Products, Munich, Germany) and compared across conditions.

In order to transform the data from the time to the time-frequency domain, the complex demodulation procedure implemented in BESA 5.2 was used. Time-frequency transformation was performed for frequencies from 4 to 100 Hz in a time window from −1000 ms to 3500 ms with respect to painful stimulation (paradigm 1) or with respect to the onset of visual stimulation (paradigm 2). Frequencies were sampled in steps of 2 Hz, latencies in steps of 25 ms. Time-frequency representations (TFR) were calculated as absolute amplitude in µV. Baseline correction was performed in MATLAB by subtracting the prestimulus interval from −1000 to 0 ms.

Analysis of neuronal responses to painful stimulation focused on three time-frequency regions of interest in the theta (4–8 Hz, 150–350 ms), alpha (8–14 Hz, 500–700 ms) and gamma frequency range (76–86 Hz, 150–350 ms), which have been recently shown to assess inter- and intraindividual differences in pain perception [24]. Additionally, FDR correction across all electrodes and the whole time-frequency range of the TFRs [28] was performed using MATLAB.

#### Paradigm 2

In paradigm 2, we assessed the effects of APTD on interference of pain with a visual attention task as a measure of cognitive aspects of pain. Reaction times to visual stimuli were registered on a trial-by-trial basis. Reaction times less than 150 ms or greater than 500 ms were excluded from further behavioral analysis. The number of excluded trials did not differ between the BAL and APTD condition (t = 1.4, p = .2). For each subject, mean reaction times to visual stimuli with (*pain* trials) and without (*no pain* trials) interfering painful stimuli in the BAL and APTD condition were calculated and compared. At the end of the experiment, we assessed the effects of APTD on unpleasantness and intensity ratings of painful stimuli as a measure of affective and sensory aspects of pain, respectively.

Time-frequency transformation of EEG data was performed as done in paradigm 1. For further details of the analysis see [26].

### Statistical Analysis

Statistical analyses of behavioral data were performed using SPSS for windows (IBM SPSS Statistics 19; IBM, Armonk, USA). Statistical analyses of neurophysiological data were performed using MATLAB. Means between conditions were compared using t-tests for paired samples. Means between conditions and times were compared using repeated measures analyses of variance (ANOVAs). To test whether conditions were associated with adverse effects, a chi-squared test was used. Correlations were calculated using Pearson’s correlation coefficient. Level of significance for hypothesis testing was p<0.05.

## Results

### Acute Phenylalanine and Tyrosine Depletion (APTD)

Amino acid levels confirmed a relative deficit of dopamine precursors and their brain availability in the APTD as compared to the BAL condition. Two two-way repeated measures ANOVAs showed a main effect of timepoint (tyrosine F_[1,21]_ = 75.9, phenylalanine F_[1,21]_ = 118.1, all p<.001) and condition (tyrosine F_[1,21]_ = 61.0, phenylalanine F_[1,21]_ = 68.0, all p<.001) for serum levels of tyrosine and phenylalanine, respectively. Most importantly, a significant condition × timepoint interaction indicates that these effects were more pronounced for the APTD than the BAL condition (tyrosine F_[1,21]_ = 95.7, phenylalanine F_[1,21]_ = 211.5, all p<.001). Post hoc t-tests confirmed that APTD treatment yielded a significant decrease in serum levels of tyrosine (M ± SD; −78±6%) and phenylalanine (−72±11%) compared to morning baselines (t≥26.4, p<.001). Ingestion of the balanced amino acid (BAL) mixture caused a slight increase of levels (tyrosine +6±37%, t = −.9, p = .4; phenylalanine +19±22%, t = −3.9, p = .001). Serum levels after APTD treatment were significantly lower than after BAL ingestion (tyrosine: t = 9.2, p<.001; phenylalanine: t = 11.6, p<.001). In each subject, the APTD mixture yielded stronger decreases of tyrosine and phenylalanine than the BAL mixture.

To determine the relative deficit of dopamine precursor availability, we calculated the ratio of tyrosine and phenylalanine to the long neutral amino acids (LNAAs: tyrosine, phenylalanine, isoleucine, leucine, methionine, tryptophan, valine). Two two-way repeated measures ANOVAs showed a main effect of timepoint (tyrosine F_[1,21]_ = 520.7, phenylalanine F_[1,21]_ = 624.8, all p<.001) and condition (tyrosine F_[1,21]_ = 61.0, phenylalanine F_[1,21]_ = 68.0, all p<.001) for the ratios of tyrosine and phenylalanine to LNAAs. Post hoc t-Tests confirmed that both APTD and BAL treatment significantly decreased ratios of phenylalanine and tyrosine to LNAAs (all t≥9.4, p<.001). However, as reflected by a significant timepoint × condition interaction, these effects were markedly more pronounced following the APTD than the BAL mixtures (tyrosine/LNAA −88±6% vs. −40±19%, F_[1,21]_ = 102.4, phenylalanine/LNAA −84±9% vs. −35±10%, F_[1,21]_ = 318.3, all p<.001).

Since an influence of the experimental procedure on the tryptophan/LNAA-ratio has been observed in previous studies [29], we additionally compared the ratio of tryptophan to LNAAs to rule out any influence of changed brain serotonine levels on pain perception or mood. A one-way repeated measures ANOVA did not show a significant main effect of timepoint (F_[1,21]_ = 1.4, p = .25) or condition (F_[1,21]_ = 2.3, p = .14).

Adverse effects of the experimental procedure included mild diarrhea and transient nausea. Three subjects reported mild diarrhea approximately 90 minutes post ingestion that could not be related to one particular AA mixture. Mild nausea was reported by eighteen subjects at 30 minutes post ingestion, by eight subjects at 4 hours post ingestion, and by two subjects at 6 hours post ingestion. The experience of nausea could not be related to one particular amino acid at any time (χ^2^≤1.8, p≥.19). Apart from that, no further adverse effects were reported.

### Influence of APTD on Motor Functioning and Mood

In order to assess a potential influence of APTD on motor speed, subjects performed a finger tapping test. As tapping rates did not differ significantly between the APTD and BAL condition (t = .2, p = .81), motor functioning can be assumed to remain unaffected by the experimental procedures.

Mood was rated on a 16-item questionnaire with three categories (‘alertness’, ‘calmness’, ‘contentment’) at three different points in time after ingestion of the AA mixture. Three two-way repeated measures ANOVAs demonstrated no significant main effect of condition (alertness: F_[1,16]_ = 3.2, p = .1; calmness: F_[1,16]_ = .1, p = .8; contentment: F_[1,16]_ = .03, p = .9) or time (alertness: F_[2,32]_ = 2.7, p = .08; calmness: F_[2,32]_ = .5, p = .6; contentment: F_[2,32]_ = 3.4, p = .054). Moreover, the analysis did not reveal a significant condition × time interaction (alertness: F_[2,32]_ = .3, p = .8; calmness: F_[2,32]_ = 3.0, p = .06; contentment: F_[2,32]_ = .04, p = 1.0), indicating that the scores in each of the three categories did not differ significantly between the APTD and BAL condition at any time.

### Paradigm 1

**Figure 2:**
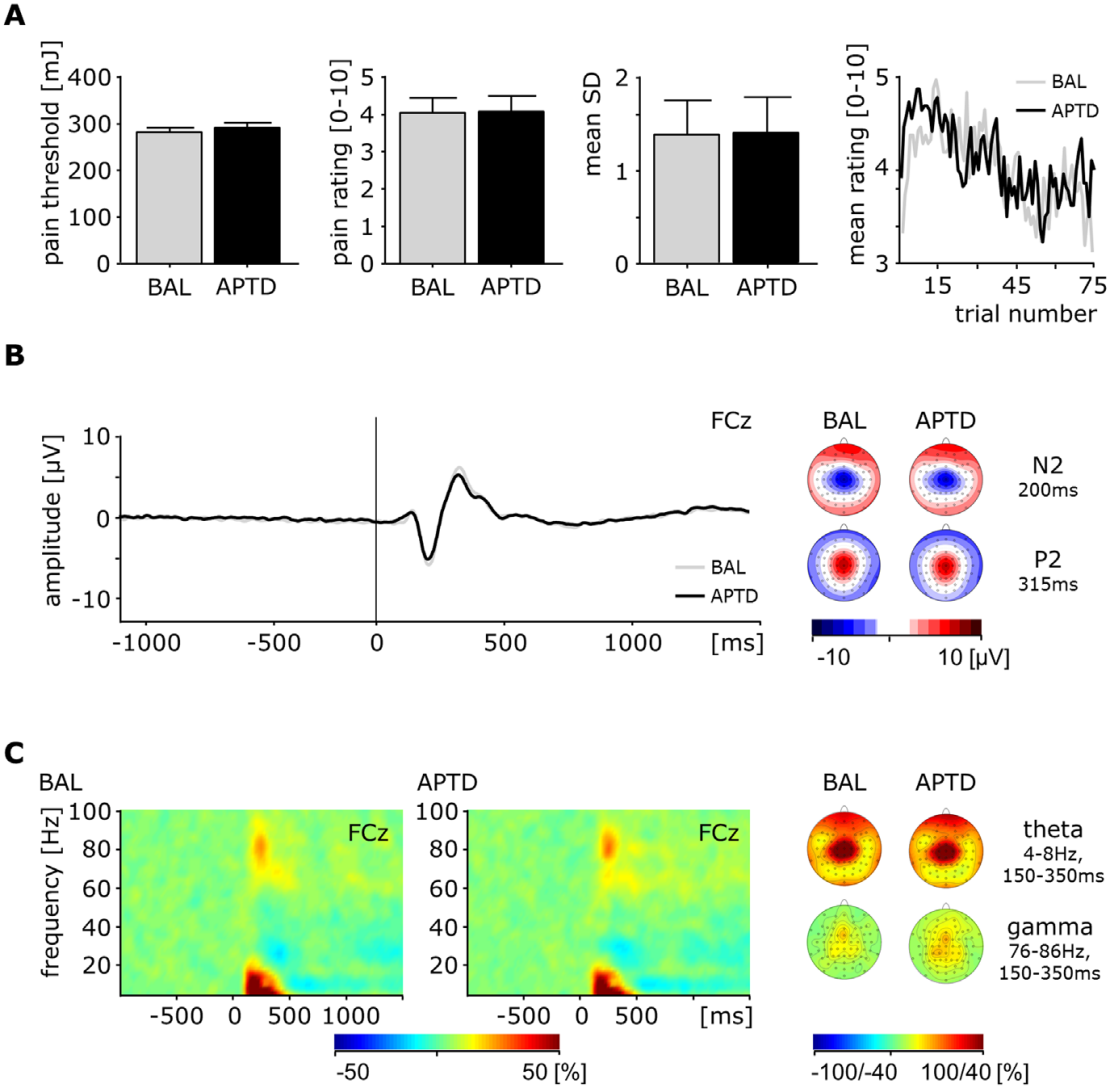
APTD does not alter pain sensation and neuronal responses to painful stimuli. (**A**) Pain thresholds, mean pain ratings, standard deviations of pain ratings, and time courses of pain ratings in the control (BAL) and depletion (APTD) condition. (**B**) Left, pain evoked potential at electrode FCz in the control and depletion condition. Right, scalp distribution of neuronal activity at 200 and 315 ms after painful stimulation in the control and depletion condition. (**C**) Left, group mean time-frequency representations of % signal change at electrode FCz after painful stimulation in the control and depletion condition. Right, scalp distribution of theta and gamma activity following painful stimulation coded as % signal change in the control and depletion condition.

In order to assess the effects of transiently reduced cerebral dopamine levels on sensory aspects of pain, we compared pain thresholds and mean values, variability and time courses of single trial pain ratings between conditions (Fig. 2A). Pain thresholds did not differ significantly between the APTD and BAL condition (291±53 vs. 283±42 mJ, respectively; t = .7, p = .47). No significant difference was found between the mean pain ratings in the APTD (4.1±2.0) versus the BAL (4.0±1.9) condition (t = .2, p = .85). Variability of pain ratings did not differ either between conditions (t = .3, p = .8). The comparison of the slopes of regression lines yielded no significant difference in habituation between the APTD and BAL condition (t = .2, p = .8).

To evaluate the effects of APTD on cerebral responses to pain, we compared laser-evoked potentials (LEP) as well as time-frequency transformed data between conditions. Painful stimulation yielded characteristic LEPs [30],[31] in both the APTD and BAL condition (Fig. 2B). Responses were most prominent over vertex electrodes with a maximum negative deflection at approximately 180 ms (corresponding to the N2 component) and a maximum positive deflection at approximately 310 ms (corresponding to the P2 component). Comparison of LEP across all electrodes and all time points post baseline did not reveal a significant difference between conditions (FDR corrected p>.05). Moreover, comparison of N2P2-peak-to-peak amplitudes did not reveal a significant difference between conditions (t = .1, p = .91). Additionally, we correlated the depletion-induced change in N2P2-peak-to-peak amplitude with the depletion-induced change in pain intensity and pain unpleasantness ratings, respectively. In neither case we found a significant correlation (pain intensity: r = .1, p = .59; pain unpleasantness: r = .01, p = .95).

Time-frequency analysis showed that painful laser stimulation yielded significant changes of neuronal activity in the previously described ROIs (Fig. 2C). Compared to a prestimulus baseline we found significant increases of theta activity (t_min_ = 7.1, p_max_ = <.001) and gamma activity (t_min_ = 5.72, p_max_ = <.001), as well as significant decreases of alpha activity (t_min_ = −5.25, p_max_ = <.001). However, pain-induced changes in neuronal activity did not differ significantly between conditions, neither in the predefined ROIs, nor considering the whole time-frequency range (FDR-corrected p>.05).

### Paradigm 2

**Figure 3:**
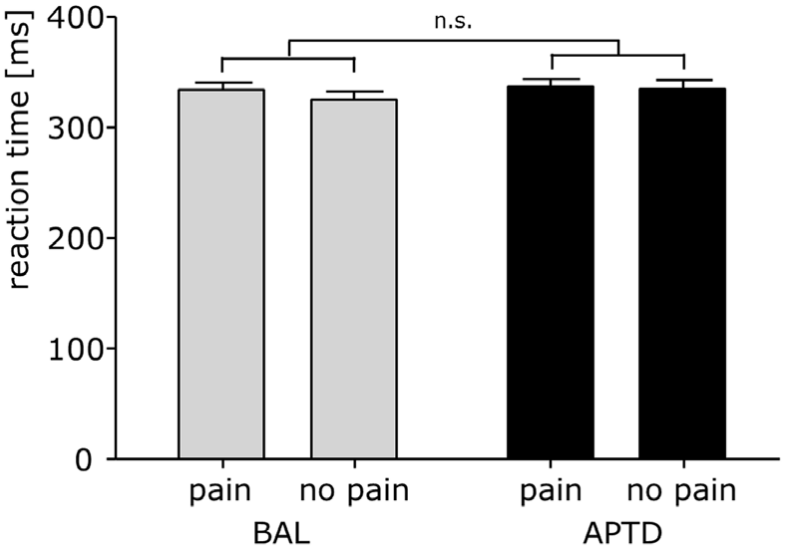
APTD does not alter pain-induced attentional interference. Reaction times to a visual stimulus in the depletion (APTD) vs. the control (BAL) condition, and in the *pain* vs. *no pain* condition, respectively.

In order to assess the effects of transiently reduced cerebral dopamine levels on cognitive aspects of pain perception, we compared the interference of pain with a visual attention task between APTD and BAL conditions. To this end, we compared pain-induced changes in reaction times (RT*_no pain_* vs. RT*_pain_*) between conditions (Fig. 3). A two-way repeated measures ANOVA with two within subjects factors demonstrated a significant main effect of stimulation (*pain* vs. *no pain*; F_[1,21]_ = 9.2, p = .006), but no significant main effect of condition (APTD vs. BAL; F_[1,21]_ = 2.8, p = .1). Most importantly, the analysis did not reveal a significant stimulation × condition interaction (F_[1,21]_ = 1.8, p = .2). Thus, the effects of pain on reaction times did not differ significantly between the APTD and BAL condition.

Time-frequency analysis showed an increase of gamma oscillations at occipital electrodes, which was centred around 60 Hz and lasted for the whole period of visual stimulus presentation (58–64 Hz, 100–2500 ms, p<.001 in both conditions). Between conditions, the strength of visually induced gamma oscillations did not differ significantly (p = .33). Interfering painful stimuli yielded an increase of gamma oscillations at central electrodes (75–200 ms, 34–64 Hz, p = .09). Between conditions, the strength of pain-induced gamma oscillations did not differ significantly (p = .46).

Next, we compared the effects of interfering painful stimuli on visual gamma oscillations between conditions. A two-way repeated measures ANOVA demonstrated no significant main effect of stimulation (*pain* vs. *no pain*; F_[1,18]_ = 2.1, p = .2) or condition (APTD vs. BAL; F_[1,18]_ = .8, p = .4). Most importantly, the analysis did not reveal a significant stimulation × condition interaction (F_[1,18]_ = .6, p = .4). Thus, the effects of interfering painful stimuli on visual gamma oscillations did not differ significantly between the APTD and BAL condition.

**Figure 4:**
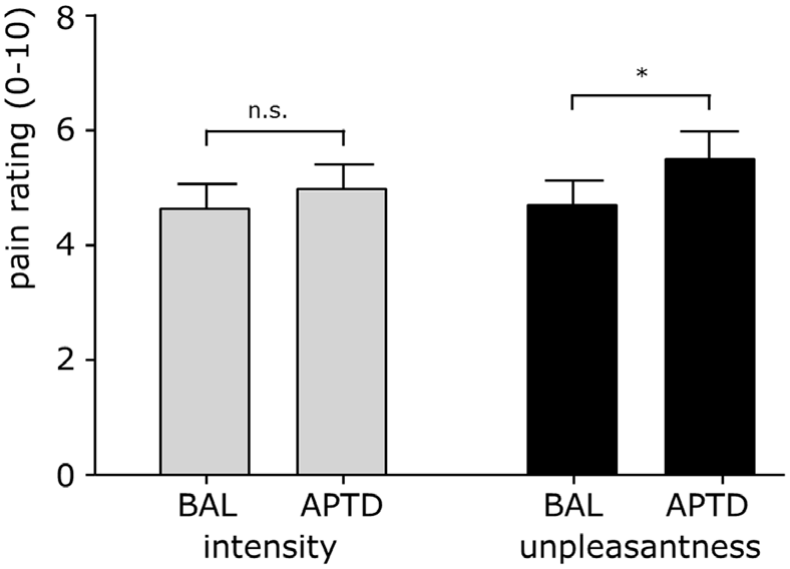
APTD increases unpleasantness, but not intensity ratings of pain. BAL  =  control condition; APTD  =  depletion condition; *, p<.05.

**Figure 5:**
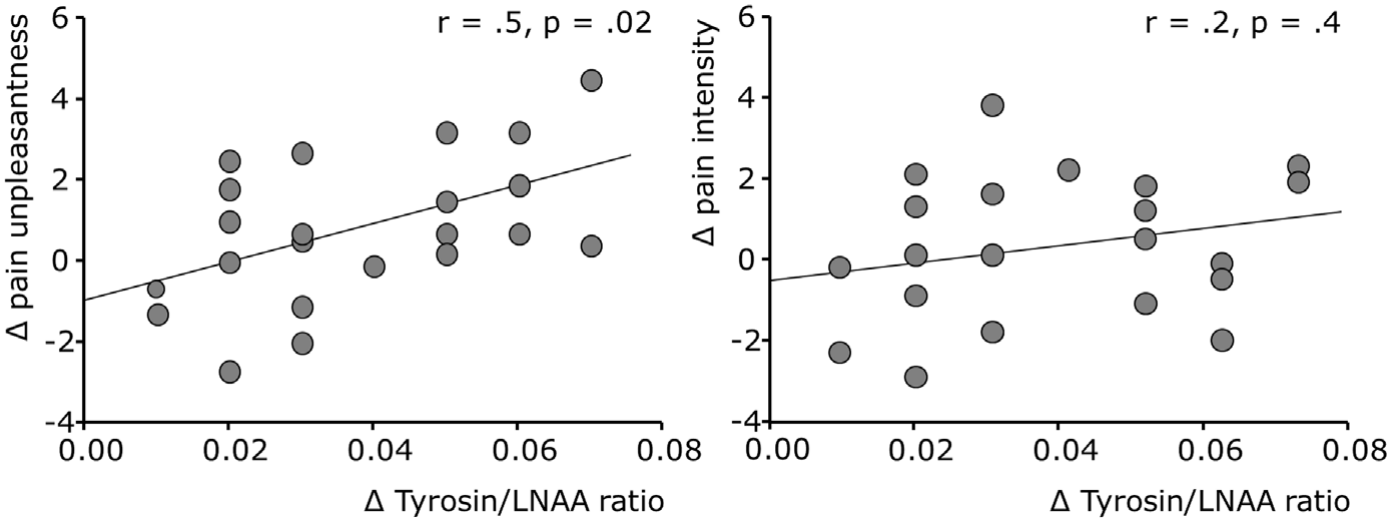
Changes in unpleasantness ratings, but not intensity ratings of pain correlate with the efficiency of the depletion procedure. Correlation between the depletion-induced decreases in the tyrosine/LNAA-ratio, and the increases of unpleasantness (left) and intensity (right) ratings under APTD.

We next compared unpleasantness and intensity ratings of pain between conditions (Fig. 4). Mean unpleasantness of painful stimulation was rated significantly higher under APTD compared to BAL condition after completion of the attention task (5.6±2.3 vs. 4.7±2.0; t = 2.1, p = .048). In contrast, neither objective stimulus intensity nor subjective ratings of pain intensity differed significantly between conditions (t = 1.2, p = .38 and t = .9, p = .23, respectively). Finally, we related the APTD-induced change in unpleasantness ratings with the APTD-induced changes of the tyrosine/LNAA-ratio (Fig. 5). The results show a positive correlation of both measures (r = .501, p = .018) indicating that subjects with a stronger depletion effect had a larger increase in unpleasantness ratings. No such correlation was found for depletion-induced decreases of phenylalanine (r = .2, p = .37). Moreover, no such correlation was found for the non-significant changes in pain intensity ratings and changes in tyrosine/LNAA-ratio (r = .2, p = .31; Fig. 5) or phenylalanine/LNAA-ratio (r = .2, p = .4), respectively.

## Discussion

We investigated the influence of dopaminergic (DA) neurotransmission on different aspects of pain perception and pain processing in the human brain. Under acute DA precursor depletion (APTD) subjects rated unpleasantness of painful stimuli significantly higher than in a control condition (BAL). Moreover, changes of unpleasantness ratings positively correlated with the effectiveness of the depletion treatment. In contrast, pain intensity ratings and neuronal responses to pain did not differ between conditions. These findings indicate a selective influence of DA neurotransmission on pain affect, whereas pain sensation remained largely unchanged.

### Dopamine and Sensory Aspects of Pain

In the present study, APTD did not modulate single trial pain intensity ratings nor their variability or the associated brain responses. APTD represents a well-established and non-invasive procedure to transiently change cerebral dopamine levels in healthy human subjects [21]. It limits potential confounds caused by comorbidity and involvement of other transmitter systems in patients suffering from DA-related diseases. The assessment of single trial pain intensity ratings was taken as a sensitive measure of pain sensation which can detect subtle changes of pain intensity as well as short-term fluctuations and changes in intensity ratings in the sense of habituation.

Our observation of unchanged pain sensation after a transient decrease of cerebral dopamine levels is in accordance with the findings of a study investigating both the effects of APTD as well as of the D2-receptor antagonist sulpiride [32]. The results of the study show that neither APTD nor sulpiride had a significant effect on pain perception, and do therefore not support the hypothesis of a direct anti-nociceptive effect of dopamine in acute experimental pain. Other studies on the relationship between DA and pain sensation yielded conflicting results. Positron emission tomography (PET) studies in healthy human subjects showed a negative correlation between baseline DA activity in the basal ganglia and an individual’s pain sensitivity [33]–[36] and a positive correlation between pain-induced DA activity in the basal ganglia and intensity of experimental pain [36], [37]. Two recent studies showed that administration of the dopamine agonist apomorphine enhanced the conditioned pain modulation [38] and led to a decrease followed by a genetically associated increase in cold pain tolerance [39] in healthy volunteers. Studies in patients with Parkinson’s disease (PD) found in some [8], [9], [40]–[42] but not all [43] cases increases of pain sensitivity in PD patients. Moreover, in some studies, the administration of L-Dopa changed pain sensitivity [8], [41], whereas in others L-Dopa did not [40], [42]. Correspondingly, significantly higher [9] as well as lower [42], [44] amplitudes of brain responses to pain were observed in PD patients as compared to healthy controls. Changes of response amplitudes were attenuated [9] or unchanged [42], [44] after administration of L-Dopa. The disparity of observations including the present one may be due to several methodological differences. First, the majority of studies did not differentially assess pain sensation and pain affect. Second, some studies investigated interindividual differences in regional DA activity whereas others assessed global intraindividual changes in DA activity under different conditions. Third, the studies partly investigated the effects of pain on DA activity and partly the effects of DA on pain, which likely represent different processes at different time scales [45]. Fourth, studies in patients might have been confounded by changes in transmitter systems other than DA [42], [44]. In this context, our observation that APTD does not influence pain sensation does not preclude any effect of dopamine on pain sensation but suggests that dopamine modulates pain affect rather than pain sensation.

### Dopamine and Affective Aspects of Pain

Our results show that painful stimuli are experienced as significantly more unpleasant, but not intense, when the cerebral dopamine levels are transiently decreased. As current measures of mood did not differ significantly between the BAL and APTD conditions, it can be assumed that this effect is not attributable to changes in mood. DA-specificity of these findings is supported by a positive correlation between increases in pain unpleasantness and decreases in cerebral DA availability under APTD.

A role for DA in pain affect has been suggested by the large anatomical and functional overlap between DA rich brain areas and areas involved in the processing of pain affect [2], [20]. The insula and anterior cingulate cortex (ACC) in particular have been shown to receive mesolimbic DA projections as well as to be involved in the affective aspects of pain [2], [20]. Furthermore, studies in experimental animals indicated that DA signalling in the insula and the ACC attenuates pain related behavior [46], [47]. A PET study in healthy humans showed that during painful stimulation striatal dopamine release was positively correlated with ratings of pain affect [36]. Moreover, a recent study in PD patients showed that administration of L-Dopa did not affect intensity but unpleasantness ratings of experimental heat pain [43]. However, the authors observed an unexpected increase of unpleasantness ratings whereas, based on the present and previous investigations [36], we would have expected a decrease of pain unpleasantness after L-Dopa. Again, the disparity of results may be due to methodological differences discussed above.

We observed APTD-induced changes of pain affect but not of pain sensation and pain-related neuronal responses. The dissociation of pain affect and pain-related neuronal responses suggests that changes of pain affect may be subserved by higher level processes, which are typically characterized by later, more distributed and less time-locked occurrence and, thus, may not be captured by event-related analyses of EEG data. The finding of unchanged electrophysiological responses suggests that other neurophysiological and -imaging methods might be better suited to identify the neuronal mechanisms subserving the selective increase of unpleasantness under APTD. At present, one can only speculate about the mechanisms underlying a selective influence of APTD on pain affect. Dopamine has consistently been shown to play a role in the motivation to obtain reward [48]–[50]. Recent evidence has linked pain and reward processing in the human brain [51], [52], suggesting that DA might be part of a “common currency for emotion” covering the range between pleasure and pain. Rather than having direct antinociceptive effects, dopamine may, thus, influence the motivation to endure or avoid pain, respectively [32]. In turn, it appears feasible that this bias towards enduring or avoiding pain is subserved by a modulation of the affective component of pain perception, while the sensory-discriminative aspect remains unaffected.

### Dopamine and Cognitive Aspects of Pain

Finally, we assessed whether APTD had an influence on an individual’s ability to attend and react to a visual stimulus during concurrent painful stimulation. Experimental and clinical observations indicate an influence of dopamine on the internal control of attentional resources as well as in the ability to shift or maintain a mental set [53]. We therefore expected an increase of pain-induced attentional interference under APTD, which was, however, not observed. As the present paradigm assesses only a partial aspect of pain-related cognitive processes, the lack of an effect of APTD on pain-induced attentional interference does not argue against a general relevance of dopamine for cognitive aspects of pain.

### Limitations

Several limitations apply to the interpretation of the present findings. First, in paradigm 1, we obtained single trial ratings of pain intensity but not of pain unpleasantness. In contrast, in paradigm 2, we assessed pain unpleasantness and pain intensity as post-hoc ratings. However, in both paradigms pain intensity ratings did not differ between the APTD and control condition indicating that APTD modulates pain affect more than pain sensation. It is nevertheless important to note that our study assesses exemplary aspects of pain sensation, cognition and affect but not the full range of pain-induced modulations of cognition and affect. Second, we applied phasic experimental pain stimuli. There is evidence that the influence of DA on pain perception differs for tonic and phasic pain [3]. Thus, the results of the present investigation apply to phasic pain, but do not necessarily hold true for tonic pain. Third, APTD may also affect the common catecholamine synthesis. It is therefore difficult to rule out collateral influence on other catecholamine neurotransmitters or precursors (e.g. tryptophane). However, there is evidence for absence of significant effects on catecholamine neurotransmitters other than DA [21].

Fourth, an effect of APTD-induced changes of mood on the observed results cannot be excluded entirely, as we did not assess baseline mood ratings prior to the experimental procedure.

Fifth, dopamine is involved in many processes at different time scales [45]. Our findings therefore apply to changes of dopaminergic signalling at the time scale of the APTD procedure but may not generalize to dopaminergic processes at all time scales.

Sixth, as the participating subjects were all males, the extrapolation of evidence from this study to women would need further evaluation.

### Conclusions

The present results, obtained with painful laser stimulation during recording of EEG, indicate that a transient decrease of cerebral dopaminergic activity in healthy human subjects modulates pain affect but not pain sensation. The differential DA effects on pain sensation and pain affect suggest that analgesic effects of dopamine might be mediated by indirect effects on pain affect rather than by direct effects on ascending nociceptive signals. These observations contribute to the understanding of the complex relationship between dopamine and pain perception. Moreover, as dopaminergic neurotransmission has been implicated in the pathology of various chronic pain states, our findings may help to understand the cerebral mechanisms and the therapy of chronic pain.
